# Lateral incision 1-stage urethroplasty with oral mucosal graft for patients with penile urethral stricture after hypospadias repair—a preliminary report

**DOI:** 10.1186/s12894-023-01250-5

**Published:** 2023-04-28

**Authors:** Jianwei Wang, Xiao Xu, Zhengqing Bao, Zhenhua Liu, Guizhong Li, Feng He

**Affiliations:** Department of Urology, Beijing Jishuitan Hospital, The Fourth Medical College of Peking University, No.68 Huinanbei Road, Changping District, Beijing, China

**Keywords:** Urethroplasty, Urethral stricture, Urethral reconstruction, Strictures after hypospadias, Hypospadias complications

## Abstract

**Purpose:**

To report our early experience of a novel surgical approach for penile urethral strictures after hypospadias repair, using a lateral incision to keep the ventral tissue and vasculature of the penis intact and to avoid the need for tissue interposition.

**Patients and methods:**

A total of 21 patients underwent lateral incision 1-stage urethroplasty with oral mucosal graft. The median age of the patients was 21 years old (range, 13–47). The median number of prior procedures for hypospadias repair was 3 (range, 1–9) with 18 of 21 patients (85.7%) undergoing greater than 1 prior reconstructive procedure. The mean length of the penile urethral strictures was 4.5 ± 1.7 cm, with a range of 1.0 to 8.0 cm. Selection criteria for lateral incision 1-stage urethroplasty include: non-obliterative stricture, no or mild penile curvature and no urethrocutaneous fistula.

**Results:**

Median follow-up was 30 months (range, 6–73). Success was achieved in 17 of 21 patients (80.9%). The 4 (19.0%) patients with treatment failure developed recurrent urethral strictures. Of the 4 men with recurrent strictures, 3 were ultimately treated successfully by DVIU (2) or two-stage urethroplasty (1), and one patient chose repeated dilation.

**Conclusions:**

For patients with penile urethral stricture after hypospadias repair with non-obliterative stricture, no significant penile curvature and no urethrocutaneous fistula, a lateral approach with oral mucosal graft is a simple technique that avoids the need for tissue interposition and keeps the penile ventral tissue and vasculature intact, resulting in a low risk of complications.

## Introduction

Urethral stricture is a common complication of prior hypospadias repair and is a challenging surgical problem for reconstructive urologists [1, 2]. Many men with penile urethral strictures resulting from failed hypospadias repair benefit from staged urethroplasty [3–5]. However, single-stage repair using buccal mucosal graft (BMG) is a viable option for patients with non-obliterative strictures [6, 7]. Conventionally, a ventral incision is performed and requires interposition of dartos fascia or tunica vaginalis as urethral coverage to reduce complications such as urethrocutaneous fistula or recurrent urethral stricture [4, 8−10].

We make a preliminary report of a new surgical approach—the lateral incision 1-stage urethroplasty with oral mucosal graft for patients with penile urethral stricture after hypospadias repair. The lateral approach is relatively simple and avoids the need for tissue interposition and preserves the ventral penile tissue. Clinical outcomes for the lateral approach appear to be comparable to the conventional ventral approach.

## Materials and methods

### Patients

Following institutional review board approval, we performed a retrospective chart analysis of patients with penile urethral stricture after hypospadias repair who underwent lateral incision 1-stage urethroplasty with oral mucosal graft. We identified 21 patients between June 2016 and July 2020. The patients ranged in age from 13 to 47, with a median of 21. The median number of prior procedures for hypospadias repair was 3 (range, 1–9) with 18 of 21 patients (85.7%) undergoing more than 1 prior reconstructive procedure. Scarred penile shaft skin with altered vascularization were common in the patients. Five (23.8%) patients underwent prior DVUI and 14(66.7%) had been managed by dilatation. Most men required multiple endoscopic dilations or urethrotomy procedures after failed repair. The original types of hypospadias were sub-coronal in 2 of the 21 patients (9.5%), mid-shaft in 12 (57.1%) and penoscrotal in 7 (33.3%). The mean length of the penile urethral strictures was 4.5 ± 1.7 ccm with a range of 1.0 to 8.0 cm. Four patients (19.0%) had persistent coronal hypospadias and two (9.5%) patients had isolated strictures of the bulbar urethra (0.5 and 1 cm). Nineteen patients underwent urethroplasty with BMG and two with labial mucosa.

Selection criteria for lateral incision 1-stage urethroplasty includes: nonobliterative stricture, no or mild penile curvature and no urethrocutaneous fistula. Preoperative evaluation included clinical history, physical examination, urine culture, residual urine measurement, uroflowmetry. All patients underwent cystoscopy and retrograde urethrography with or without voiding cystography.

### Surgical technique

**Fig. 1:**
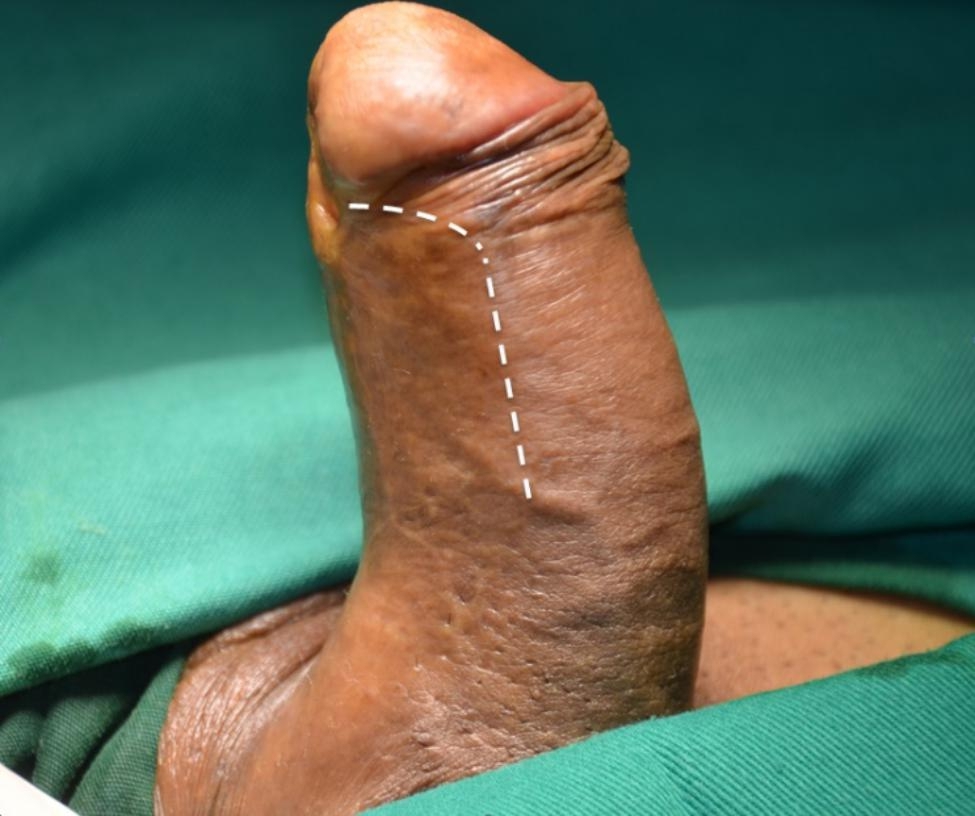
The lateral incision. Dashed line shows the incision along the left side of the penis, which can be extended distally towards the corona, and then medially toward the meatus along the coronal sulcus

**Fig. 2:**
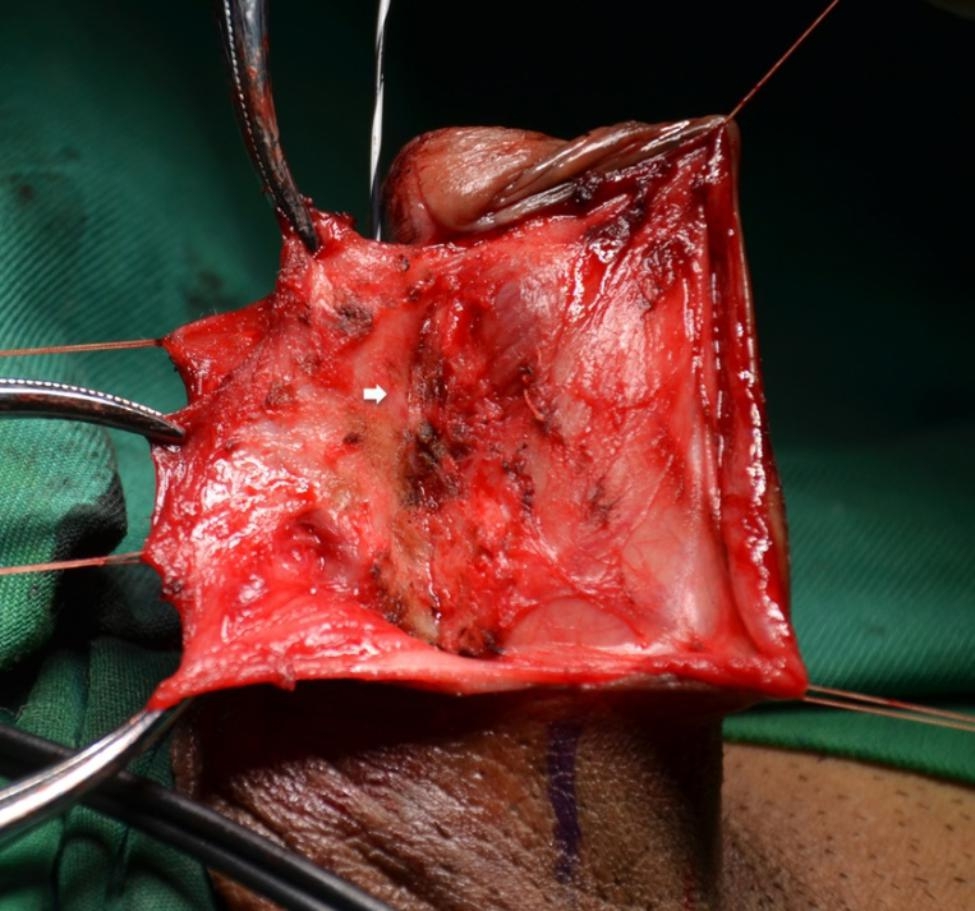
Dissection of the urethral plate off the underlying tunica albuginea. White arrow shows the dorsal aspect of the urethra

The patient is placed in a supine position. The skin of the lower abdomen and genitalia is shaved and this region is prepared and draped appropriately. A lateral longitudinal incision along the left side of the penile shaft is made. If the fossa navicularis is involved, the incision should be elongated distally towards the corona and then medially along the coronal sulcus toward the meatus if necessary (Fig. 1). The penile skin and underlying dartos fascia are dissected along the surface of Buck‘s fascia until the urethra is reached. Further dissection is required to separate the urethral plate from the underlying tunica albuginea of the corpora cavernosa which is on the dorsal side of the stricture segment (Fig. 2). The urethra is partially rotated with stay sutures and the distal extent of the stenosis is identified by retrogradely inserting a urethral catheter.

**Fig. 3:**
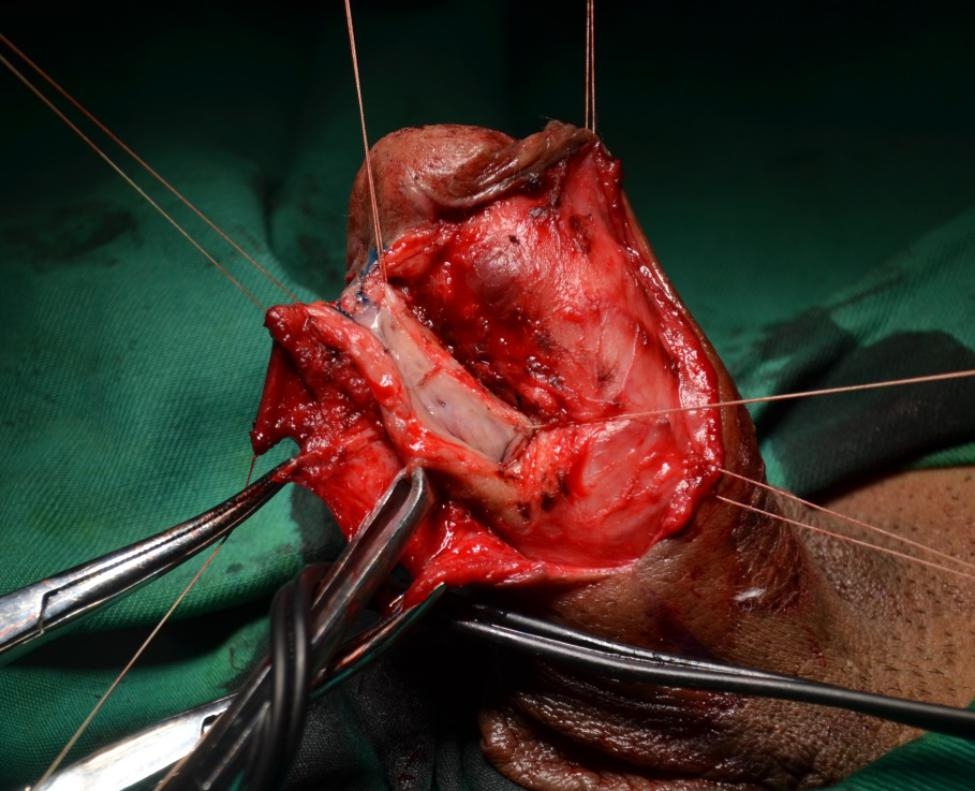
The stricture is opened dorsally

**Fig. 4:**
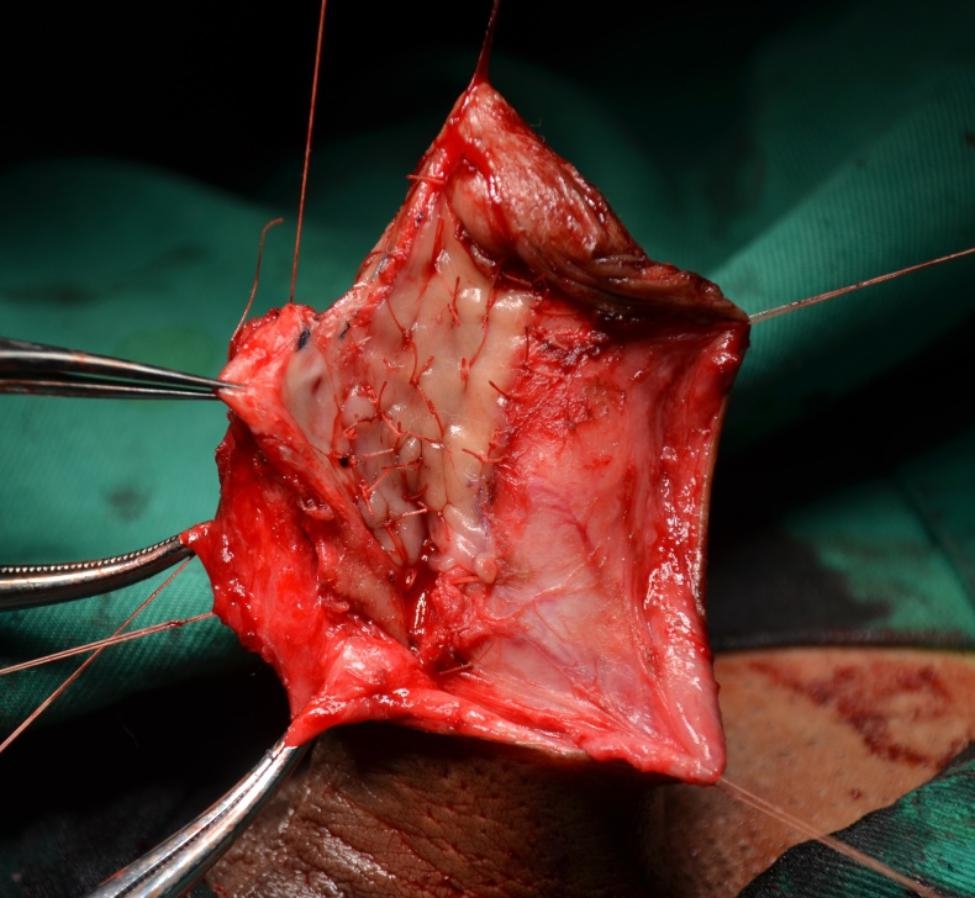
Oral mucosa graft is fixed over the tunica albuginea

**Fig. 5:**
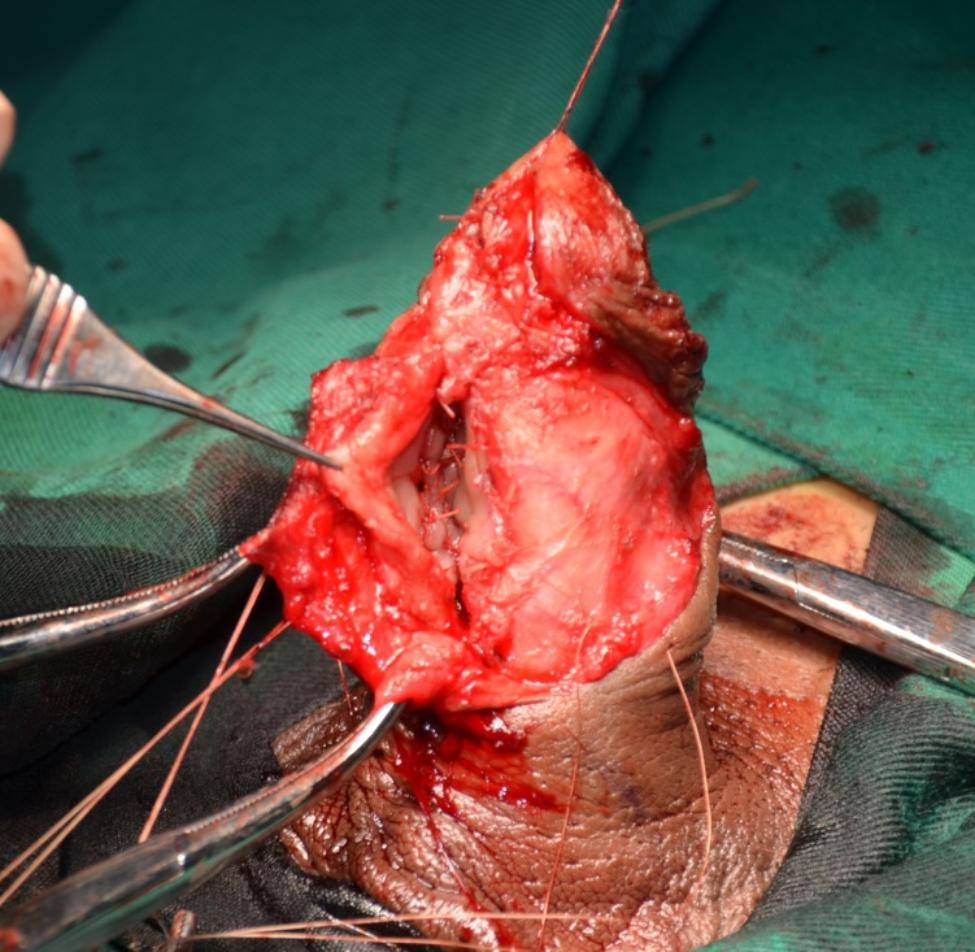
Stabilizing the urethral margins over the graft

**Fig. 6:**
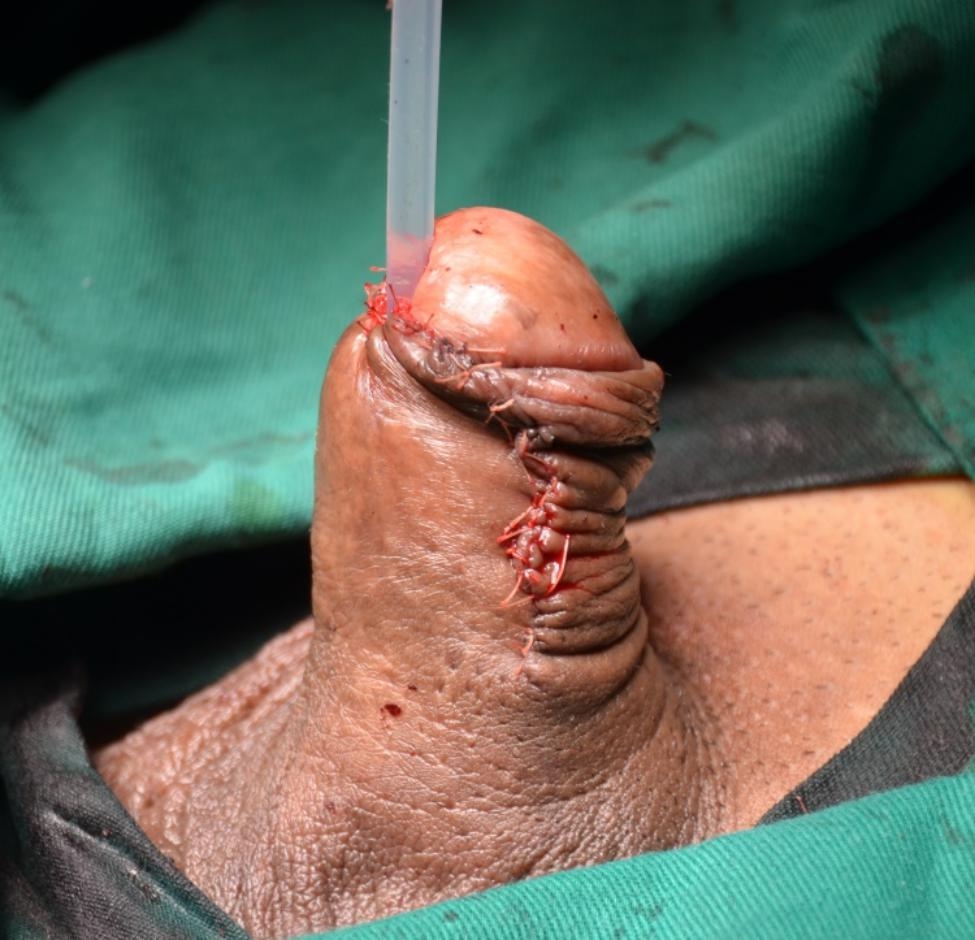
Sutured lateral incision maintaining the ventral tissue of the penis intact

The urethra is opened dorsally distal to the stricture, and is then incised proximally through the stricture and beyond a short distance, laying the stricture entirely open. (Figure 3). Once the entire stricture has been incised, the length and width of the remaining urethral plate is measured. The oral mucosal graft is harvested from the cheek or inner lower lip in a standard fashion. The oral mucosal graft is trimmed to an appropriate size and spread fixed over the tunica albuginea (Fig. 4). The two apices of the graft are sutured to the proximal and distal apices of the urethrotomy. Both the distal and proximal ends of the graft should enter well into the normal mucosa of the urethra beyond the stricture. The right margin of the oral graft is sutured to the left margin of the urethral plate. A Foley 14 F silicone catheter is inserted (F10 for paediatric patients). Interrupted sutures are used to stabilize the urethral margins over the graft on the left side (Fig. 5). At the end of the procedure, the graft is completely covered by the urethra and the dartos fascia while maintaining the ventral tissue of the urethra intact (Fig. 6).

Two patients with concomitant bulbar urethral strictures underwent simultaneous non-transecting bulbar urethroplasty, in order to protect the impaired vasculature of the corpus spongiosum [11]. This was performed in lithotomy via an additional perineal incision.

### Follow-up

We removed the urethral catheter 2 weeks later. Success was defined as the patient having no obstructive symptoms and requiring no further treatment. Patients were followed up postoperatively at 3, 6, and 12 months and annually thereafter. Voiding cystourethrogram was performed at catheter removal and retrograde urethrogram were obtained when subjective obstructive symptoms were present or the possibility of any complication was suspected.

## Results

Median follow-up was 30 months, with a range of 6 to 73 months. Initial success was achieved in 17 of 21 patients (80.9%). In the 4 (19.0%) patients in whom treatment failed, this was due to recurrent stricture. There were no cases of urethrocutaneous fistula or diverticulum and no patient experienced skin necrosis due to lateral incision. Of the 4 men in whom surgery failed with recurrent strictures 3 were ultimately treated successfully by DVIU (2) of two-stage urethroplasty (1), and the remaining patient elected repeated dilation. There was no clinical evidence of fistula or wound dehiscence at last follow up in all 21 patients. Two patients (9.5%) claimed the decrease of penile sensitivity.

## Discussion

Despite advances in the technique of hypospadias repair, some patients have complications and may require multiple surgeries during childhood, adolescence and even into adulthood. Thus, innate deficiencies of the corpus spongiosum and multiple failed operations makes further management challenging [1–3, 12]. Urethral strictures after hypospadias repair are the leading cause of iatrogenic urethral stricture in men younger than 45 years [13]. The most common site of secondary urethral strictures after childhood hypospadias repair is the penile urethra [2, 12]. There is no rule for therapeutic dilations to manage urethral strictures in children and repeat visual urethrotomy has not been effective [7, 14, 15]. Open re-do urethroplasty has been recommended for all recurrent strictures complicating hypospadias repair, even those still less than 1 cm [7]. Excision with anastomosis for urethral strictures after hypospadias repair is not recommended due to the fact that the vascularity of the neourethra is not reliable and shortening of the penile urethra will lead to the occurrence of chordee [16]. The choice of 1-stage or multistage repair remains controversial and is mostly based on surgeon preference and experience, whether in pediatric or adult patients [3].

Although some reported series prefer two-stage urethroplasty, for well-selected patients with a sufficient urethral plate, one-stage urethroplasty may represent the best option, with relatively high failure-free results [1, 2, 12]. Treatment of adults with complications from previous hypospadias surgery, in which most of them were urethral stricture, showed that repair with a 1-stage procedure with a penile skin flap fared the worst, with a 75% complication rate [1]. However, in patients who have a non-obliterative stricture and a healthy residual urethral plate, single-stage repair using graft, especially oral mucosal graft, is a viable option and preferred [6, 7]. Traditionally, non-obliterative strictures after hypospadias repair can be corrected with a 1-stage augmented urethroplasty with a ventral midline incision made in the raphe as degloving the penis is not necessary and can bring more trauma to the impaired vasculature of the local tissue.

Besides urethral stricture, urethrocutaneous fistula is one of the commonest complications reported after hypospadias repair, with a wide variability in reported incidence (0–28%) [17]. To reduce the incidence of fistula occurrence, interposition techniques using de-epithelised skin, spongiosum, dartos flap or tunica vaginalis flap have been used to cover the neourethra with variable results [18]. Although dartos and tunica vaginalis flaps are most frequently used as the interposition layers to prevent fistula formation, the preparation of those flaps is complex, which can extend operation time and increase the risk of complications. After multiple surgeries. the penile skin is often scarred and lacks a normal dependable vascular supply, which also makes the dartos flap preparation challenging. Also, dartos flap interposition has been associated with torsion and ischemia of the penile skin, whereas tunica vaginalis interposition may lead to chordee and risks injury of testis and its vascularity [19, 20].

Thus, local vasculature protection is a key issue during the process of reconstruction. Lateral incision rather than median ventral incision can preserve or protect the ventral penile vasculature and blood supply while completing a one-stage repair. Therefore, a lateral approach avoids the common complications associated with a traditional ventral incision because the ventral penile tissue and vasculature remain intact. Furthermore, the lateral incision technique avoids the need for interposition flap preparation, and thus simplifies the procedure. Although managing problems from previous hypospadias surgery is difficult with a high initial failure rate [1], there were no cases of urethrocutaneous fistula or diverticulum in our series.

After multiple surgeries the penile skin and urethra are often scarred and lack a normal dependable vascular supply and patients who have undergone prior urethroplasty may have skin or urethra that are based on unknown and unpredictable vascular pedicles [1, 4]. So, the occurrence of urethral stricture and fistula may result from ischemia and necrosis of the molding material used for urethral reconstruction [21]. Furthermore, the research of Manzoni et al. showed that complications after hypospadias repair such as urethral stricture could develop with genital maturity in some patients, even in those with apparently excellent outcomes during childhood [22]. Another explanation was that urethral strictures after hypospadias repair were new problems and not associated with lingering complications from their childhood surgery and the presentation of adults having strictures of the neourethra after childhood repair indicated that these repairs might not be durable through puberty and the onset of sexual activity, especially when there was weak or absent corpus spongiosum [7]. However, Snodgrass [5] investigated the development of complications after childhood hypospadias repair and nearly all his patients reported urethral complications before puberty, so those hypospadias complications in adults might represent complications never corrected after childhood surgery. Barbagli et al. [10] suggest that in patients with hypospadias it was the congenital lack of spongiosum tissue that promotes the urethral deterioration over time as the skin-made urethra did not tolerate the repeated stretch and trauma during erection and sexual activity as otherwise normal spongiosum-made urethra. Actually, the structure and flexible texture of spongiosum might act as a cushion between the external forces and the urethra while protecting it during erection and sexual activity. Above all, the exact reasons leading to the formation of urethral stricture after hypospadias repair needs further investigation.

In conclusion, non-obliterative penile urethral strictures with relatively healthy urethral plate are probably best treated by lateral incision 1-stage urethroplasty with oral mucosal graft. The traditional ventral approach to this problem involves local tissue trauma and further impaired local vasculature, as well as requiring tissue interposition which makes the urethroplasty more complicated and increases the risk of complications. Therefore, there are theoretical and practical reasons why a ventral approach might best be avoided in favour of a lateral approach. We have devised a simple procedure that avoids damage to the ventral soft tissue of the urethra, and which seems to be at least as effective as traditional 1-stage graft inlay. However, we must interpret the results of this investigation with caution because patient selection was very stringent, only including non-obliterative strictures, with no or mild penile curvature and no urethrocutaneous fistula.

## Conclusions

For patients with penile urethral stricture after hypospadias repair with non-obliterative stricture, no significant penile curvature and no urethrocutaneous fistula, a lateral approach with oral mucosal graft is a simple technique that avoids the need for tissue interposition and keeps the penile ventral tissue and vasculature intact, resulting in a low risk of complications.

## Data Availability

The datasets used and/or analysed during the current study available from the corresponding author on reasonable request.

## Abbreviations

BMG: Buccal Mucosal Graft
DVIU: Direct Vision Internal Urethrotomy
